# Tumor heterogeneity evaluated by computed tomography detects muscle-invasive upper tract urothelial carcinoma that is associated with inflammatory tumor microenvironment

**DOI:** 10.1038/s41598-021-93414-2

**Published:** 2021-07-09

**Authors:** Keisuke Goto, Yukiko Honda, Kenichiro Ikeda, Kenshiro Takemoto, Toru Higaki, Tetsutaro Hayashi, Kohei Kobatake, Yuko Nakamura, Yohei Sekino, Shogo Inoue, Kazuo Awai, Wataru Yasui, Jun Teishima

**Affiliations:** 1 Department of Urology, Graduate School of Biomedical and Health Sciences, Hiroshima University, 1-2-3 Kasumi, Minami-ku, Hiroshima, 734-8551 Japan; 2 Department of Diagnostic Radiology, Graduate School of Biomedical and Health Sciences, Hiroshima University, Hiroshima, Japan; 3 Department of Molecular Pathology, Graduate School of Biomedical and Health Sciences, Hiroshima University, Hiroshima, Japan

**Keywords:** Urology, Ureter

## Abstract

To detect muscle-invasive upper tract urothelial carcinoma, we evaluated the internal texture of the tumor using texture analysis of computed tomography images in 86 cases of upper tract urothelial carcinoma. The internal texture of the tumor was evaluated as the value of computed tomography attenuation number of the unenhanced image, and the median, standard deviation, skewness and kurtosis were calculated. Each parameter was compared with clinicopathological factors, and their associations with postoperative prognosis were investigated. Immunohistochemistry was performed to investigate the histological and molecular mechanisms of the inflammatory tumor microenvironment. The histogram of computed tomography attenuation number in non-muscle invasive tumor was single-peaked, whereas muscle invasive tumor showed a multi-peaked shape. In the parameters obtained by texture analysis, standard deviation was significantly associated with pathological stage (*p* < 0.0001), tumor grade (*p* = 0.0053), lymphovascular invasion (*p* = 0.0078) and concomitant carcinoma in situ (*p* = 0.0177) along with recurrence-free (*p* = 0.0191) and overall survival (*p* = 0.0184). The standard deviation value correlated with the amount of stromal components (*p* < 0.0001) and number of tumor-infiltrating macrophages (*p* < 0.0001). In addition, higher expression of high mobility group box 1 was found in heterogeneous tumor. Tumor heterogeneity evaluated by texture analysis was associated with muscle-invasive upper tract urothelial carcinoma and represented an inflammatory tumor microenvironment and useful as the clinical assessment to differentiate muscle invasive tumor.

## Introduction

Upper tract urothelial carcinoma (UTUC), arising from the renal pelvis and ureter, is a relatively rare malignancy that accounts for only 5–10% of urothelial carcinomas, with an estimated incidence of approximately 2 cases per 100,000 person-years^1^. Despite recent improvements in imaging and endoscopic techniques that have led to stage migration toward an earlier stage, UTUC still represents an aggressive disease with high rates of recurrence and progression. Several lines of studies suggested the pathological parameters of tumor grade, T stage, lymphovascular invasion (LVI) and concomitant carcinoma in situ (CIS) to be prognostic factors in UTUC^2^. Although the pathological findings are useful to estimate survival prognosis, these parameters are available only after surgery. Recently, some evidence has suggested that neoadjuvant chemotherapy (NAC) offers therapeutic benefits to patients with UTUC^3^. It would be preferable if the pathological parameters could be estimated preoperatively because these findings might allow better determination of the indications for NAC in UTUC. However, criteria for the identification of patients indicated for NAC are still being explored because preoperative T categorization of UTUC has not been well established. Previously, it was reported that the computed tomography (CT) image pattern could clearly differentiate T stage according to the shape of the tumor mass and spicula^4^. However, there were some discrepancies between CT estimation and the microscopic stage. To explain these discrepancies, we suspected that the internal texture might be different between non-muscle invasive and muscle invasive tumor.

Texture analysis (TA) is a novel imaging tool to measure tissue heterogeneity that depends on objective computer-aided evaluation of gray-level patterns within a lesion^5–7^. However, there might be some technical complications to be used parameters obtained by TA. In the present study, we used more simple parameters obtained from TA to evaluate the internal texture of UTUC and investigated its association with the clinicopathological parameters. In addition, we examined whether the parameters obtained from TA were associated with the prognosis of UTUC. Furthermore, we investigated the association between CT findings and histological findings of tumor microenvironment (TME) that might contribute to tumor progression in UTUC.

## Results

### Patient characteristics

We reviewed 206 patients with UTUC but without any metastases who underwent radical nephroureterectomy at Hiroshima University Hospital. Patients who received preoperative or postoperative chemotherapy or those with chronic or systemic inflammatory disease were excluded from this study. Only the patients with a single tumor lesion were included in this study so that the association between the main tumor with variables measured by TA and clinicopathological parameters and prognosis could be evaluated. Likewise, patients with prior or concomitant bladder cancer were excluded because the presence of urothelial carcinoma of the urinary bladder may affect the results of this study. Accordingly, 86 patients were included in this study (Table 1).

**Table 1:** Characteristics of the 86 patients with upper tract urothelial carcinoma.

Age, years (median, range)	73 (52–93)
**Sex**	
Female	18
Male	68
**Location**	
Ureteropelvic junction	32
Ureter	54
**Laterality**	
Left	47
Right	39
**Hydronephrosis**	
Mild	59
Severe	27
**Urine cytology**	
Negative	40
Positive	46
**T stage**	
Ta	32
T1	17
T2	7
T3	29
T4	1
**Histological grade**	
Low	29
High	57
**Lymphovascular invasion**	
Not evident	59
Evident	27
**Concomitant carcinoma in situ**	
No	63
Yes	23
Follow-up, months (median)	33.5

### TA could detect stroma-rich tumors in histology

**Figure 1:**
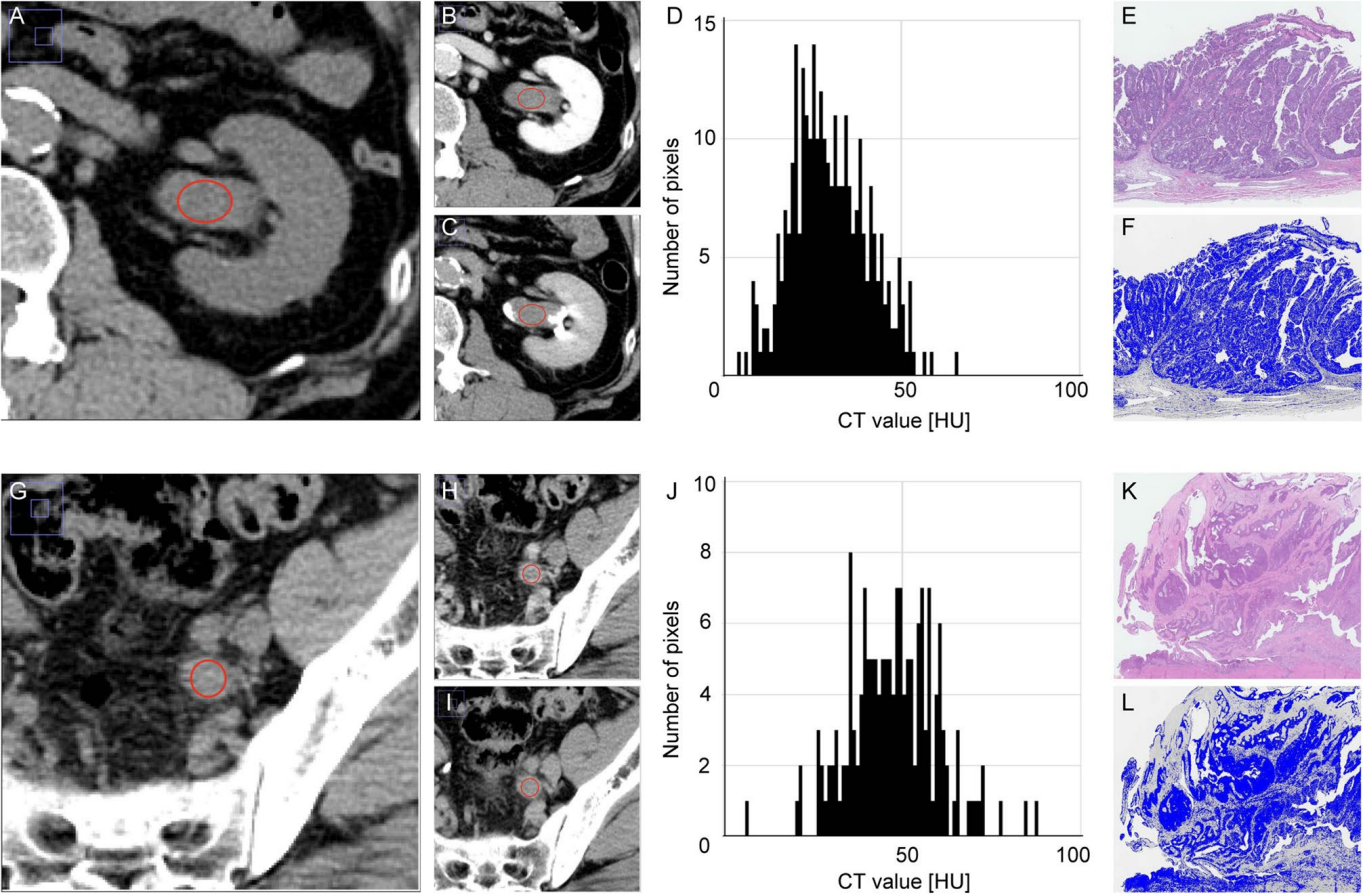
Illustration of ROIs and corresponding histological findings in representative cases of non-muscle invasive UTUC and muscle invasive UTUC. (**A**–**C**) In non-muscle invasive UTUC, unenhanced (**A**), nephrogenic (**B**) and excretory (**C**) images are shown synchronically to detect tumor in the renal pelvis, and the ROI (red oval) was determined. (**D**) The histogram of CT value in HU in the tumor revealed a shape with almost a single peak. (**E**) Hematoxylin and eosin staining showed typical non-invasive papillary urothelial carcinoma. (**F**) Image analysis to estimate the amount of stromal components in tumor tissue using Image J software. An appropriate threshold was drawn to distinguish tumor cells and stromal components. (**G**–**I**) In muscle invasive UTUC, the ROI was determined with unenhanced (**G**), nephrogenic (**H**) and excretory (**I**) images. (**J**) The histogram revealed a shape with a broad base and multiple peaks. (**K**) Hematoxylin and eosin staining showed muscle invasive urothelial carcinoma. (**L**) Image analysis using Image J software. ROI = region of interest, UTUC = upper tract urothelial carcinoma, CT = computed tomography, HU = Hounsfield unit, SD = standard deviation, TA = texture analysis.

In 86 cases of UTUC, the region of interest (ROI) was determined from axial image and then the value of the CT attenuation number was measured in Hounsfield units (HU). The histogram of each indicated ROI was drawn, and the median CT value, standard deviation (SD), skewness and kurtosis were calculated as the parameters. The cases with non-muscle invasive UTUC, the CT image revealed a tumor mass with smooth border (Fig. 1A–C). The histogram of the tumor revealed a shape with almost a single peak (Fig. 1D). Histologically, a papillary architecture of carcinoma cells with a fibrovascular core was evident, and the tumor was diagnosed as non-invasive papillary urothelial carcinoma (Fig. 1E). When an appropriate threshold was drawn to distinguish carcinoma cells and stroma, fewer stromal components were shown in the tumor (Fig. 1F). In the cases with muscle invasive UTUC, the CT image revealed a tumor mass with heterogeneous texture and irregular border (Fig. 1G–I). The shape of the histogram was broad based and multi-peaked (Fig. 1J). The tumor invaded into the muscular layer and was diagnosed as invasive urothelial carcinoma (Fig. 1K). When distinguishing the stromal components, more stromal components were evident (Fig. 1L).

### Parameters obtained from TA are associated with histological findings

**Figure 2:**
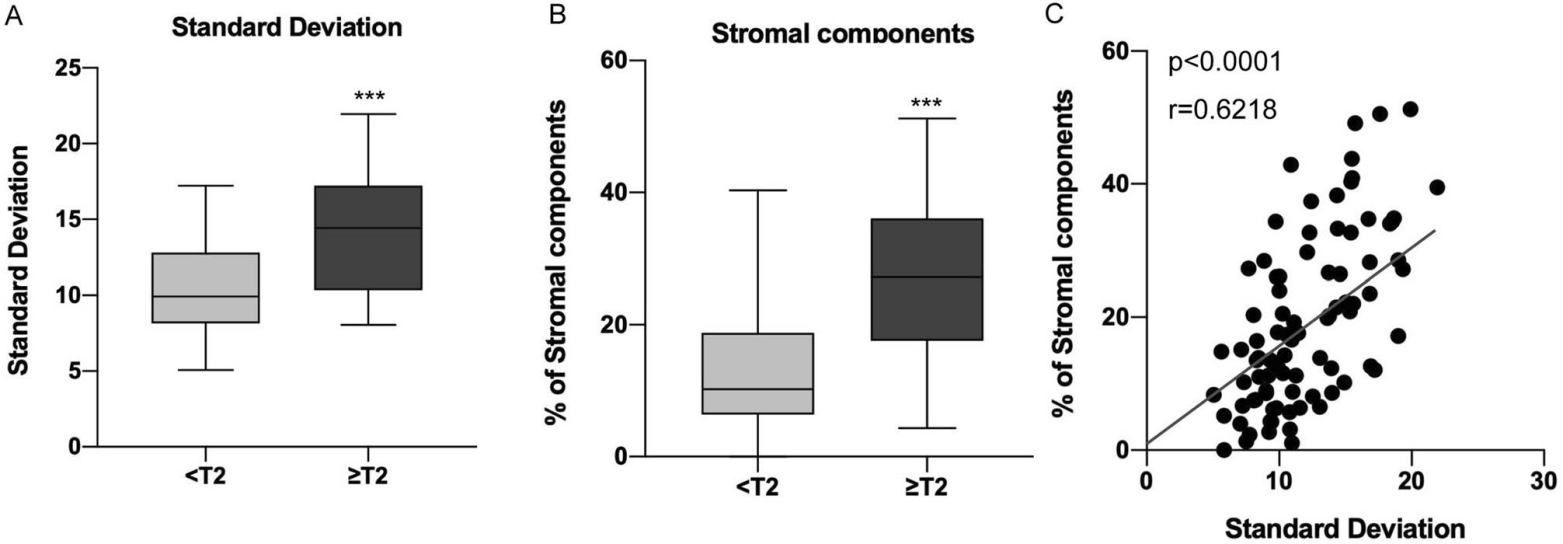
The associations between SD and histological findings. (**A**) Boxplot for SD obtained from TA according to pathological T stage. (**B**) Boxplot for the amount of stromal components according to pathological T stage. (**C**) Scatter plot comparing SD in TA and the amount of stromal components in histology. ***p < 0.0001. SD = standard deviation, TA = texture analysis.

Next, we investigated whether the variables obtained from TA were associated with clinicopathological factors (Table 2). Among the parameters, SD was significantly associated with pathological T stage (*p* < 0.0001, Fig. 2A), histological grade (*p* = 0.0053), LVI (*p* = 0.0078) and concomitant CIS (*p* = 0.0177). Also, more stromal components were present in > pT2 muscle invasive UTUC than in non-muscle invasive (pTa-T1) tumors (*p* < 0.0001, Fig. 2B). When the amount of stromal component and SD were compared, a significant correlation was found (*p* < 0.0001, Fig. 2C). These results suggested that SD obtained from TA was closely associated with histological findings in UTUC.

**Table 2:** Relationships between clinicopathological parameters and values calculated with texture analysis.

	Median CT value		Standard deviation		Skewness		Kurtosis	
	Mean ± SD	*p* value	Mean ± SD	*p* value	Mean ± SD	*p* value	Mean ± SD	*p* value
**Hydronephrosis**								
Mild	35.62 ± 5.74	**0.0035**	11.78 ± 3.72	0.4119	− 0.060 ± 0.36	0.9788	− 0.022 ± 0.40	0.3018
Severe	39.11 ± 4.52		12.56 ± 4.21		− 0.061 ± 0.27		− 0.109 ± 0.34	
**Urine cytology**								
Negative	36.25 ± 6.03	0.4752	11.61 ± 3.70	0.3577	− 0.053 ± 0.32	0.8343	− 0.071 ± 0.33	0.6241
Positive	37.13 ± 5.23		12.38 ± 4.02		− 0.068 ± 0.35		− 0.031 ± 0.43	
**T stage**								
Ta-T1	35.51 ± 5.56	**0.0196**	10.50 ± 3.14	** < 0.0001**	− 0.038 ± 0.33	0.4613	− 0.086 ± 0.34	0.3286
T2-T4	38.32 ± 5.31		14.03 ± 3.86		− 0.092 ± 0.34		− 0.000 ± 0.43	
**Histological grade**								
Low	35.65 ± 4.47	0.1683	10.48 ± 3.33	**0.0053**	− 0.023 ± 0.33	0.4566	− 0.118 ± 0.29	0.1860
High	37.26 ± 6.06		12.81 ± 3.92		− 0.080 ± 0.33		− 0.014 ± 0.42	
**Lymphovascular invasion**								
Not evident	35.75 ± 5.64	**0.0128**	11.28 ± 3.75	**0.0078**	− 0.070 ± 0.32	0.7194	− 0.076 ± 0.35	0.3899
Evident	38.85 ± 4.99		13.66 ± 3.68		− 0.040 ± 0.36		0.010 ± 0.44	
**Concomitant CIS**								
No	36.36 ± 5.83	0.2977	11.42 ± 3.77	**0.0177**	− 0.044 ± 0.31	0.5160	− 0.128 ± 0.35	**0.0032**
Yes	37.70 ± 4.93		13.67 ± 3.72		− 0.105 ± 0.40		0.160 ± 0.38	

*CT* computed tomography, *SD *standard deviation, *CIS* carcinoma in situ.

The significance of bold means *p* < 0.05.

### TA parameters predict tumor invasiveness preoperatively

To suggest a better way to differentiate the indications for NAC, we investigated the significance of parameters from TA as potential predictors for invasive UTUC by logistic regression modeling (Table 3). In the univariate analysis, hydronephrosis (odds ratio [OR] = 3.667, *p* = 0.0071), urine cytology (OR = 4.714, *p* = 0.0013), median CT value (OR = 2.478, *p* = 0.0481) and SD (OR = 5.914, *p* = 0.0002) were significantly associated with higher T stage. In the multivariate analysis, hydronephrosis (OR = 4.838, *p* = 0.0094), urine cytology (OR = 7.729, *p* = 0.0005) and SD (OR = 9.963, *p* = 0.0001) were the independent predictors of higher T stage. Taken together, SD values obtained from TA were useful to assess clinical stage and hydronephrosis and urine cytology, which are already known as important predictors of high risk for UTUC.

**Table 3:** Univariate and multivariate analysis for predicting higher T stage according to various clinical parameters.

	Univariate analysis			Multivariate analysis		
	OR	95% CI	*p* value	OR	95% CI	*p* value
**Hydronephrosis**						
Mild	1 (Ref.)			1 (Ref.)		
Severe	3.667	1.421–9.788	**0.0071**	4.838	1.462–17.96	**0.0094**
**Urine cytology**						
Negative	1 (Ref.)			1 (Ref.)		
Positive	4.714	1.804–13.59	**0.0013**	7.729	2.358–30.62	**0.0005**
**Median CT value (HU)**						
Low	1 (Ref.)			1 (Ref.)		
High	2.478	1.008–6.335	**0.0481**	1.287	0.396–4.129	0.6705
**Standard deviation**						
Low	1 (Ref.)			1 (Ref.)		
High	5.914	2.249–17.20	**0.0002**	9.963	2.959–40.94	**0.0001**
**Skewness**						
Low	1 (Ref.)			1 (Ref.)		
High	1.240	0.508–3.040	0.6351	0.847	0.250–2.752	0.7831
**Kurtosis**						
Low	1 (Ref.)			1 (Ref.)		
High	0.940	0.383–2.290	0.891	0.837	0.250–2.723	0.7669

*OR* odds ratio, *CI* confidence interval, *CT* computed tomography, *HU* Hounsfield unit.

The significance of bold means *p* < 0.05.

### SD values obtained from TA was associated with worse prognosis in UTUC

**Figure 3:**
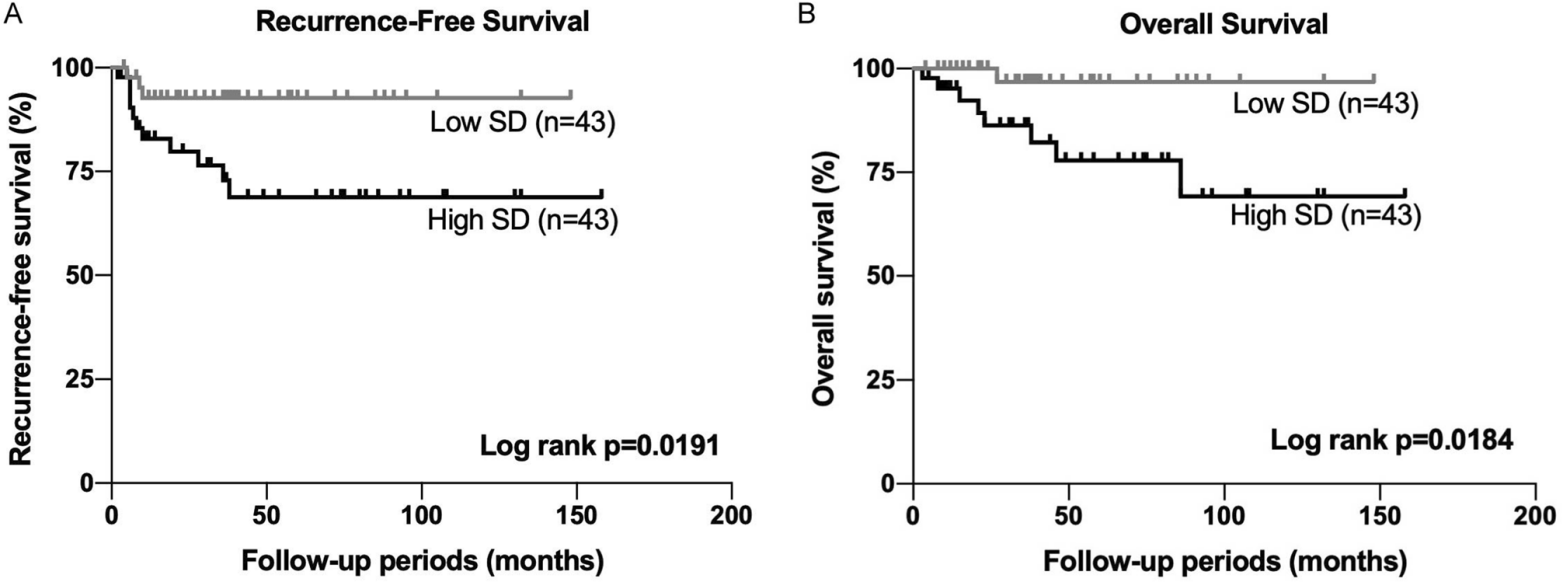
The survival analysis according to SD in UTUC. (**A**) Kaplan–Meier analysis for RFS according to SD. (**B**) Kaplan–Meier analysis for OS according to SD. SD = standard deviation, UTUC = upper tract urothelial carcinoma, TA = texture analysis, RFS = recurrence-free survival, OS = overall survival.

To determine the clinical significance of SD value as the predictive factor in UTUC, the association between SD and clinical outcome was examined by Kaplan–Meier analysis. The patients with higher SD had significantly worse prognosis both for RFS (*p* = 0.0191, Fig. 3A) and OS (*p* = 0.0184, Fig. 3B). However, no significant associations were found in the other parameters (Fig. S1). Furthermore, when the univariate and multivariate Cox proportional hazard models were used (Table 4), SD was the independent predictor for both RFS (HR = 3.533, *p* = 0.0489) and OS (HR = 8.415, *p* = 0.0179). Together with these results, SD measured with TA was associated with worse prognosis, suggesting that tumor heterogeneity could have important roles in the aggressiveness of UTUC.

**Table 4:** Univariate and multivariate cox proportional hazard model for RFS and OS according to clinical parameters.

	Univariate analysis			Multivariate analysis		
	HR	95% CI	*p* value	HR	95% CI	*p* value
**Recurrence free survival**						
Hydronephrosis						
Mild	1 (Ref.)			1 (Ref.)		
Severe	1.158	0.388–3.457	0.7928	1.091	0.317–3.387	0.8835
Urine cytology						
Negative	1 (Ref.)			1 (Ref.)		
Positive	3.505	1.092–15.52	**0.0341**	2.894	0.843–13.39	0.094
Median CT value (HU)						
Low	1 (Ref.)			1 (Ref.)		
High	1.44	0.500–4.380	0.4978	1.032	0.319–3.486	0.9577
Standard deviation						
Low	1 (Ref.)			1 (Ref.)		
High	4.072	1.271–18.01	**0.0167**	3.533	1.066–16.42	**0.0489**
Skewness						
Low	1 (Ref.)			1 (Ref.)		
High	3.211	1.069–11.75	**0.0373**	2.175	0.658–8.550	0.2085
Kurtosis						
Low	1 (Ref.)			1 (Ref.)		
High	0.98	0.342–2.810	0.9697	1.09	0.347–3.304	0.9031
**Overall survival**						
Hydronephrosis						
Mild	1 (Ref.)			1 (Ref.)		
Severe	1.042	0.313–3.469	0.9459	1.091	0.317–3.387	0.8835
Urine cytology						
Negative	1 (Ref.)			1 (Ref.)		
Positive	1.521	0.482–5.170	0.6576	2.643	0.582–14.50	0.2112
Median CT value (HU)						
Low	1 (Ref.)			1 (Ref.)		
High	1.198	0.381–4.062	0.7579	1.332	0.297–7.198	0.7131
Standard deviation						
Low	1 (Ref.)			1 (Ref.)		
High	4.895	1.286–31.89	**0.0177**	8.415	1.384–162.0	**0.0179**
Skewness						
Low	1 (Ref.)			1 (Ref.)		
High	1.11	0.271–4.272	0.8787	0.695	0.149–3.165	0.6332
Kurtosis						
Low	1 (Ref.)			1 (Ref.)		
High	0.428	0.428–7.786	0.48	1.217	0.288–6.264	0.7946

*HR* hazard ratio, *CI* confidence interval, *CT* computed tomography, *HU* Hounsfield unit.

The significance of bold means *p* < 0.05.

### Macrophage infiltration is associated with muscle invasive UTUC

Based on the results that SD values obtained from TA were significantly associated with tumor invasiveness and prognosis, this suggested that tumor heterogeneity determined by TA might represent a more aggressive tumor. To explore this interesting supposition, we investigated the backgrounds of the invasive UTUC with a statistical algorithm using a public database (Fig. S2). Because actual mRNA expression profiles were not available in our 86 cases of UTUC, the alternative dataset deposited in public database was used for an analysis. Then we used the xCell algorithm that is based on tumor mRNA data and predicts the levels of 64 comprehensive cell types in tumor. When the xCell score was estimated in 32 cases of UTUC, the immune score was significantly higher in muscle invasive UTUC than in non-muscle invasive tumor (*p* = 0.0481). Furthermore, the macrophage score was also significantly higher in muscle invasive tumors (*p* = 0.0274). Therefore, we focused on macrophage infiltration and investigated whether tumor heterogeneity was associated with the number of infiltrating macrophages in UTUC.

### Infiltration of tumor-associated macrophages is correlated with tumor heterogeneity

**Figure 4:**
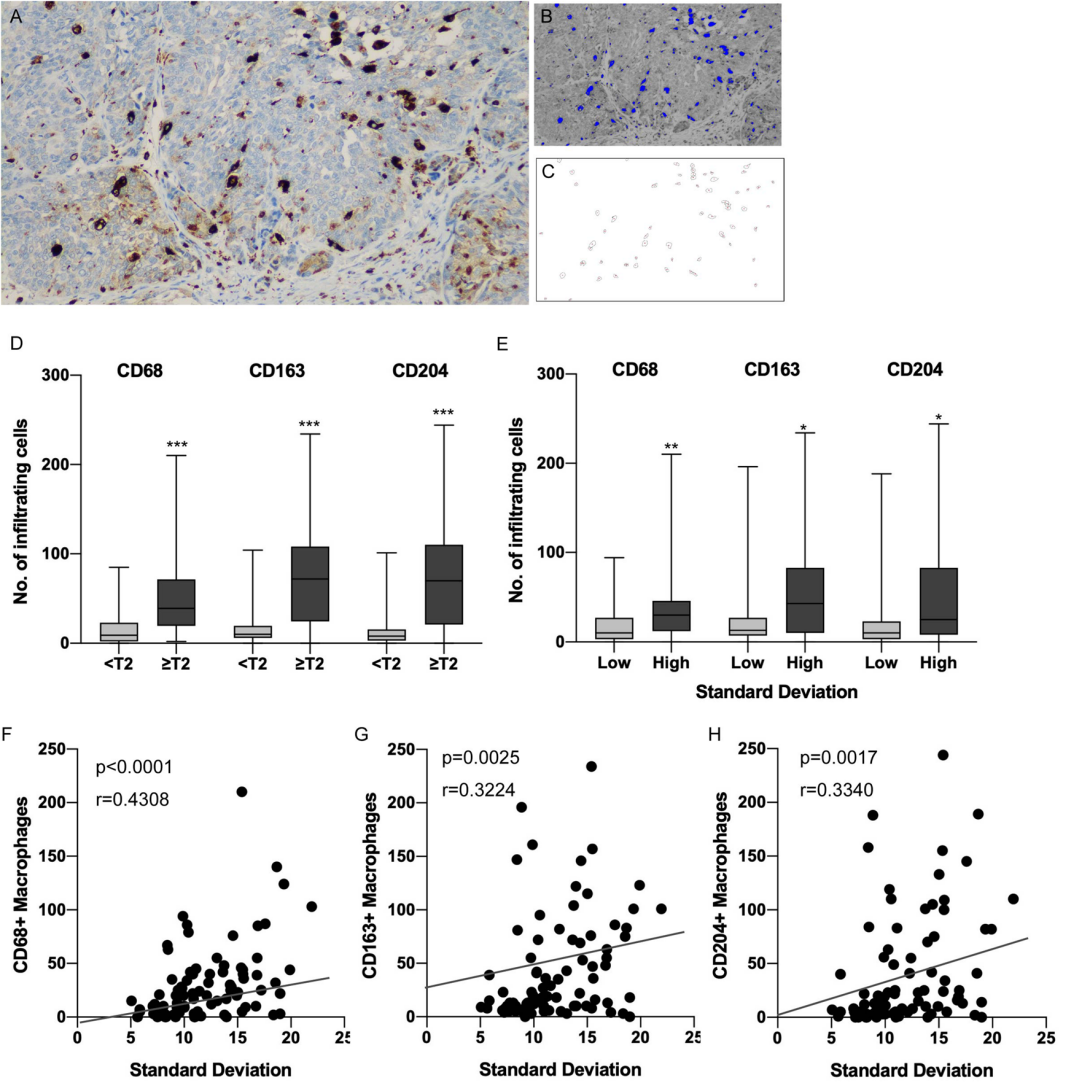
Tumors with heterogeneous internal texture in CT had more macrophage infiltration in UTUC. (**A**) Representative case of CD68 IHC that detected macrophages in muscle invasive urothelial carcinoma. (**B**) Image for detecting CD68-positive macrophages by the threshold using Image J software. Blue polygonal lesions indicate the CD68-positive macrophages. (**C**) Automatic counting of the number of infiltrating macrophages. (**D**) Boxplots for the number of infiltrating CD68-, CD163- and CD204-positive macrophages according to pathological T stage. (**E**) Boxplots for the number of infiltrating CD68-, CD163- and CD204-positive macrophages according to standard deviation obtained by TA. (**F**) Scatter plot comparing SD in TA and the number of CD68-positive macrophages. (**G**) Scatter plot comparing SD in TA and the number of CD163-positive macrophages. (**H**) Scatter plot comparing SD in TA and the number of CD204-positive macrophages. *p < 0.05, **p < 0.001, ***p < 0.0001. CT = computed tomography, UTUC = upper tract urothelial carcinoma, IHC = immunohistochemistry, TA = texture analysis.

To confirm whether the macrophage infiltration was associated with muscle invasive UTUC in our cohort, the numbers of macrophages were counted using immunohistochemistry (IHC) of CD68 to indicate every kind of macrophage (Fig. 4A). The image was converted so that the cells stained with DAB were counted at an appropriate threshold to detect IHC-positive cells (Fig. 4B,C). The numbers of CD163- and CD204-stained cells were also counted in the same way to determine tumor-associated macrophages (TAMs). When the number of macrophages was compared according to pT stage, more CD68-, CD163- and CD204-positive macrophages (all, *p* < 0.0001) had infiltrated the muscle invasive tumors than the non-muscle invasive tumors (Fig. 4D). Also, when the number of macrophages was compared according to tumor heterogeneity evaluated by TA, more CD68-, CD163- and CD204-positive macrophages (*p* = 0.0078, *p* = 0.0177 and *p* = 0.0224, respectively) had infiltrated the heterogeneous tumors (Fig. 4E). Consistently, positive correlations were found between SD in TA and the number of CD68-, CD163- and CD204-positive macrophages (*p* < 0.0001, Fig. 4F; *p* = 0.0025, Fig. 4G; and *p* = 0.0017, Fig. 4H; respectively), whereas no significant correlations were found in the other parameters from TA (Fig. S3). These results suggested that macrophage infiltration might be a part of heterogeneous component of UTUC.

### Macrophage infiltration is associated with prognosis of UTUC

To determine the clinical significance of macrophage infiltration in UTUC, we evaluated its association with clinicopathological parameters and clinical outcomes. More macrophage infiltrations were found in cases with positive urine cytology, higher T stage, higher histological grade, lymphovascular invasion and concomitant CIS (Table S1). The patients with abundant infiltration of macrophages had significantly worse prognosis for RFS and OS (Fig. S4). Because the high likelihood of positive findings could influence the results, we evaluated the relationships between SD and macrophage infiltration according to each pathological parameter (Table S2). Additional analysis suggested that T stage might be confounding positive correlations between SD and macrophage infiltration, but there was evident association between SD and macrophage infiltration in cases without LVI. These results suggested that tumor heterogeneity as evaluated by TA partially correlated with macrophage infiltration that was associated with the prognosis of UTUC.

### Cytoplasmic HMGB1 expression is associated with macrophage infiltration, tumor heterogeneity and prognosis

**Figure 5:**
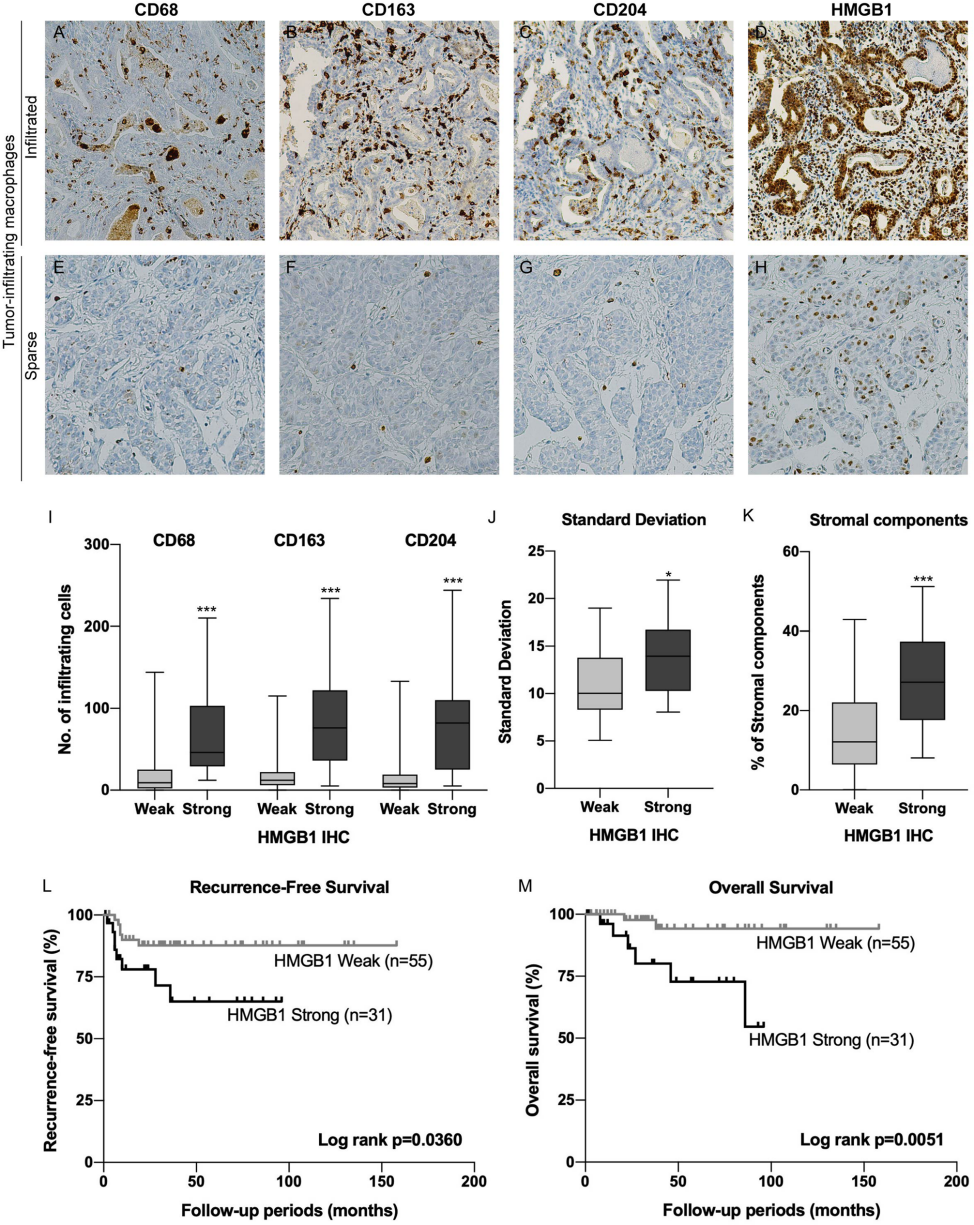
HMGB1 expression was associated with tumor heterogeneity and macrophage infiltration in UTUC. Representative cases of UTUC with abundant macrophage (**A**–**D**) and sparse macrophage infiltration (**E**–**H**). The tumor with more infiltrating CD68- (**A**), CD163- (**B**) and CD204- (**C**) positive macrophages showed strong HMGB1 expression (**D**). Conversely, the tumor with fewer CD68- (**E**), CD163- (**F**) and CD204- (**G**) positive macrophages showed weak HMGB1 expression (**H**). (**I**) Boxplots for CD68-, CD163- and CD204-positive macrophages according to HMGB1 expression. (**J**) Boxplot for SD according to HMGB1 expression. (**K**) Boxplot for the amount of stromal components according to HMGB1 expression. (**L**) Kaplan–Meier analysis for RFS according to HMGB1 expression. (**M**) Kaplan–Meier analysis for OS according to HMGB1 expression. *p < 0.05, ***p < 0.0001. HMGB1 = high mobility group box 1, UTUC = upper tract urothelial carcinoma, SD = standard deviation, RFS = recurrence-free survival, OS = overall survival.

That macrophage infiltration into the tumor was associated with tumor progression in UTUC suggested that local chronic inflammatory reactions might have important roles within the TME. To determine how macrophages were recruited to tumor tissue, we explored the associations between tumor stage and representative molecules that induce macrophages using 32 cases of UTUC available in cBioportal (http://www.cbioportal.org) and found higher expression of high mobility group box 1 (HMGB1) in muscle invasive UTUC (Fig. S5). To validate the association between HMGB1 expression and the number of tumor-infiltrating macrophages, we examined HMGB1 expression in UTUC by IHC. Abundant macrophages infiltrated tumor tissue in the cases with higher HMGB1 expression (Fig. 5A–D), whereas few infiltrating macrophages were present in cases with weak HMGB1 expression (Fig. 5E–H). When the number of macrophages were compared between the cases with weak versus strong HMGB1 expression, more CD68 (*p* < 0.0001), CD163 (*p* < 0.0001) and CD204 (*p* < 0.0001) macrophage infiltrations were found in the cases with higher HMGB1 expression (Fig. 5I). To confirm whether tumor heterogeneity evaluated by TA was associated with HMGB1 expression, we compared parameters from TA according to HMGB1 expression. The SD value was significantly higher in the cases with higher HMGB1 expression (*p* = 0.0011, Fig. 5J), whereas no significant differences were found between HMGB1 expression and the other parameters (Table S1). Consistently, more interstitial components were found in the cases with higher HMGB1 expression (*p* < 0.0001, Fig. 5K). Also, HMGB1 expression was significantly associated with pathological T stage (*p* < 0.0001), tumor grade (*p* = 0.0002), LVI (*p* = 0.0116) and concomitant CIS (*p* = 0.0184, Table 5). Furthermore, the patients with higher HMGB1 expression had significantly worse prognosis both for RFS (*p* = 0.0360, Fig. 5L) and OS (*p* = 0.0051, Fig. 5M). Taken together, these results suggested that cytoplasmic HMGB1 could recruit macrophages into tumor tissue and participate in a heterogeneous TME that was associated with worse prognosis of UTUC.

**Table 5:** Relationships between HMGB1 expression and clinicopathological parameters.

	HMGB1 IHC		*p* value
	Weak	Strong (%)	
**Hydronephrosis**			
Mild	41	18 (30.5%)	0.1169
Severe	14	13 (48.2%)	
**Urine cytology**			
Negative	28	12 (30.0%)	0.2746
Positive	12	19 (41.3%)	
**T stage**			
Ta-T1	42	7 (14.3%)	**< 0.0001**
T2-T4	13	24 (64.9%)	
**Histological grade**			
Low grade	26	3 (10.3%)	**0.0002**
High grade	29	28 (49.1%)	
**Lymphovascular invasion**			
Not evident	43	16 (27.1%)	**0.0116**
Evident	12	15 (55.6%)	
**Concomitant CIS**			
No	45	18 (28.6%)	**0.0184**
Yes	10	13 (56.5%)	

*HMGB1* high mobility group box 1, *IHC* immunohistochemistry, *CIS* carcinoma in situ.

The significance of bold means *p* < 0.05.

## Discussion

In this study, we evaluated the internal texture of UTUC using TA technique and differentiated non-muscle invasive from muscle invasive tumor by the SD value. Previously, the usefulness of some clinical factors was reported to determine high risk for UTUC. Hydronephrosis was the common clinical parameter that might be associated with a higher stage^8,9^. However, some cases might show a discrepancy with the actual stage because hydronephrosis itself could occur as a result of luminal obstruction or extrinsic compression, and it might result in tumor invasiveness being estimated indirectly. For more accurate assessment, a tumor mass should be evaluated directly by imaging examinations. CT and magnetic resonance imaging are the most common and useful examinations for a diagnosis of UTUC^10,11^. A tumor mass can be detected as a mass lesion with or without an irregular external surface on these images. It is known that multidetector-row computed tomography can differentiate organ-confined tumors and locally invasive tumors with high accuracy^12^. It has also been reported that magnetic resonance urography with diffusion-weighed imaging is applicable for UTUC staging because the apparent diffusion coefficient can be used as the objective parameter^13,14^. However, it remains unclear whether the value of the apparent diffusion coefficient can be used to distinguish non-muscle invasive UTUC from muscle invasive UTUC. In the present study, we used the value of SD obtained from TA as the parameter and showed significant associations between pathological parameters including T stage, tumor grade, lymphovascular invasion and concomitant CIS. In addition, a significant correlation between SD and the amounts of interstitial components was revealed, suggesting that TA could recognize tumors with desmoplastic change, which is an important finding in muscle invasive tumors.

In the present study, we used xCell (https://xcell.ucsf.edu/) algorithm to investigate the background of invasive UTUC in silico. The xCell scoring system integrates the gene set enrichment and provides the compendium of gene signatures for comprehensive 64 cell types including immune cells, hematopoietic cells, epithelial cells, and extracellular matrix cells derived from thousands of expression profiles to gain a better perspectives on considering tumor microenvironment^15^. When the xCell scores were estimated, the tumor stage was significantly associated with the number of macrophages and that the number of infiltrating CD68-, CD163- and CD204-positive macrophages were significantly associated with tumor heterogeneity as evaluated by TA. It is known that macrophages are directly involved in tumor progression and metastasis through their multiple functions such as inflammation, matrix remodeling, intravasation and angiogenesis^16^. Although CD68 is used as a classical marker for macrophages^17^, the plasticity of macrophages should also be considered. M1- and M2-like macrophages are two representative phenotypes of macrophages that are also called pro-inflammatory and pro-tumoral macrophages, respectively^18^. TAMs have M2-like phenotypes and play important roles in tumor by cooperating with various cytokines and chemokines^19^. CD163 and CD204 are used as markers for TAMs, and the prognostic value of macrophage polarization using M1/M2 identifying markers has been well studied in many kinds of epithelial neoplasms^20^. We showed that macrophage infiltration was significantly associated with poor prognosis in UTUC. Therefore, SD evaluated by TA might be a feasible way to predict more aggressive tumors and other factors that were suggested previously.

HMGB1 is one of the representative damage-associated molecular patterns and is strongly associated with chronic inflammation that induces macrophages^21^. HMGB1 is normally present in the nucleus of the cell and functions as a non-histone binding protein that regulates chromatin structure^22^. HMGB1 is actively secreted from macrophages or passively released from necrotic cells and acts as a proinflammatory mediator that induces cytokine production including TNF-α^23,24^ or a chemoattractant according to redox status^25^. In addition, it has been reported that HMGB1 is secreted from cancer cells and is associated with tumor growth, invasion and metastasis through its binding with several cell surface receptors including the receptor for advanced glycation end products (RAGE) and Toll-like receptors^26^. Several articles have reported HMGB1 as a prognostic factor for survival^27^. Consistently, we showed a positive correlation between HMGB1 expression and macrophage infiltration and that strong HMGB1 expression was associated with higher stage and poor prognosis in UTUC. Furthermore, we suggest that HMGB1-mediated inflammation might contribute to the production of heterogeneous tumor mass involving tumor cells, macrophages and other stromal components.

This study has a couple of limitations. Firstly, the number of cases was small. Second is that the design of the study was retrospective setting. Following to the present study, prospective and multi-center study would be required to resolve these limitations. Other limitations are regarding image analysis. In this study, three dimensional CT images were not used in all cases of this study. Because coronal or sagittal images with same condition were not available in all of the cases, the evaluation was performed only by the axial image, which was performed in all cases. Indeed, only the single axial image was useful when the small tumor was evaluated. Ideally, the segmentation should be performed in 3D to get an accurate representation of the 3D tumor. Although ROI was consensually determined by two diagnostic abdominal and urogenital radiologists, there still might be the possibility of inter- and intra-observer variability. Further consideration would be needed to provide objective systems in image analysis. Those should be performed as the following prospective study.

In summary, tumor heterogeneity evaluated by TA was associated with histological heterogeneity in UTUC and represented a HMGB1-mediated inflammatory tumor microenvironment. CT is one of common examinations that is performed to most patients with UTUC for staging. TA might be applicable to the clinical assessment to determine the criteria for NAC and useful in improving the quality of treatment for UTUC.

## Methods

### Patients

In this retrospective study, 206 patients with UTUC who underwent radical nephroureterectomy at Hiroshima University Hospital were examined. The informed consent was obtained from all of the patients that participated in this study. The relevant clinical data including hydronephrosis and urine cytology collected from the upper urinary tract were obtained from medical records. Intravesical recurrence was excluded from the survival analysis. This study was approved by the Ethics Committee of Hiroshima University (authorization number: E-589-1) and performed in accordance with relevant guidelines and regulations.

### CT examination and texture analysis

All procedures for TA were performed using Image-J software^28^. Three phases (unenhanced, nephrogenic and excretory) of the CT images were prepared. The ROI was manually drawn as an oval to include the majority of the tumor without the tumor border to avoid artifact of surrounding fat or urine. Two diagnostic abdominal and urogenital radiologists consensually set the ROI on the single axial image with the largest cross-sectional tumor for analysis. At first, the ROI was manually drawn within the tumor border on enhanced image, which the lesion was clearly shown. Image-J enabled to measure the same site by synchronizing ROI that was selected by enhanced CT on unenhanced CT. To avoid the influences of enhancement timing and artifact, the corresponding unenhanced image was used as the actual ROI for TA. In this way, the value of the CT attenuation number was measured in Hounsfield units (HU). The histogram of each indicated ROI was drawn, and the median CT value, standard deviation (SD), skewness and kurtosis were calculated as the parameters for statistical analysis. All of these parameters were evaluated without providing any other clinical information, so that it was independent of other analyses including clinical evaluation and immunohistochemical analysis.

### Immunohistochemistry

We collected formalin-fixed, paraffin-embedded tissues from the 86 patients with UTUC obtained by radical nephroureterectomy. IHC was performed with a Dako EnVision+ rabbit peroxidase detection system (Dako Cytomation, Glostrup, Denmark) as described previously^29^. Antibodies for CD68 (Kp-1, Dako), CD163 (10D6, Novocastra, Newcastle, UK), CD204 (SRA-E5, Trans Genic, Kobe, Japan) and high mobility group box 1 (HMGB1, ab18256, Abcam, Cambridge, UK) were used as the primary antibodies. Sections were stained with DAB and counterstained with 0.1% hematoxylin. The number of CD68-, CD163- or CD204-positive cells was counted automatically using Image-J software. Three representative hot spots were determined and captured as image files and converted to 8-bit images. Appropriate thresholds were drawn to highlight positive particles for each marker within each hot spot. The particles were counted automatically, and the average count of the three hot spots was calculated as the number of macrophages. The IHC scores were evaluated independently not to be influenced by TA scores.

### In silico analysis using public database

The xCell algorithm was used to investigate the backgrounds of invasive UTUC. We used 32 cases of mRNA expression data that was available in the database of Genotypes and Phenotypes (dbGaP; Accession number is phs001087.v2.p1). This dataset is derived from the manuscript described about the analysis of whole-exome and RNA sequencing of UTUC by Robinson et al.^30^ and also on cBioportal for Cancer Genomics with the identifier https://www.cbioportal.org/study?id=utuc_cornall_baylor_mdacc_2019.

### Statistical analysis

All continuous variables are shown as the average and SD. Differences in variables with continuous distribution across categorical parameters were tested using a two-tailed Student *t*-test or Mann–Whitney U test. The Pearson chi-square test was used to compare the distributions of categorical variables. A logistic regression model was used in the univariate and multivariate analysis to compare categorical parameters. For survival analysis, Kaplan–Meier survival curves were constructed for single parameter and recurrence-free survival (RFS) and overall survival (OS) as the prognosis. The log-rank test was used to evaluate the statistical significance. A Cox hazard regression model was used for univariate and multivariate analysis with estimated hazard ratio (HR) and 95% confidence interval determined. A value of *p* < 0.05 was considered as statistically significant in each comparison. All statistical analyses were performed with JMP v15.0 software (SAS Institute, Cary, NC), and the Kaplan–Meier survival curves were drawn using GraphPad Prism v8.0 software (GraphPad Software Inc., San Diego, CA).

## Supplementary Information

Supplementary Information.
