# Supplementary material for: Tumor heterogeneity evaluated by computed tomography detects muscle-invasive upper tract urothelial carcinoma that is associated with inflammatory tumor microenvironment
Source: Sci Rep. 2021 Jul 9;11:14251. doi: 10.1038/s41598-021-93414-2 (PMC8271017; doi:10.1038/s41598-021-93414-2)
Supplement: Supplementary file 1 — Supplementary Information. [file 41598_2021_93414_MOESM1_ESM.pdf]

Supplementary Information

Tumor heterogeneity evaluated by computed tomography detects muscle-invasive upper tract urothelial carcinoma that is associated with inflammatory tumor microenvironment

Goto K, Honda Y, Ikeda K, Tekemoto K, Higaki T, Hayashi T, Kobatake K, Nakamura Y, Sekino Y, Inoue S, Awai K, Yasui W and Teishima J

Supplementary figures

Figure S1
Scatter plots and survival curves according to parameters obtained from texture analysis.

Figure S2
xCell scores in 32 cases of UTUC.

Figure S3
Scatter plots according to parameters obtained from texture analysis

Figure S4
Survival curves according to the number of CD68-, CD163- and CD204- positive macrophages

Figure S5
mRNA expressions in 32 cases of UTUC.

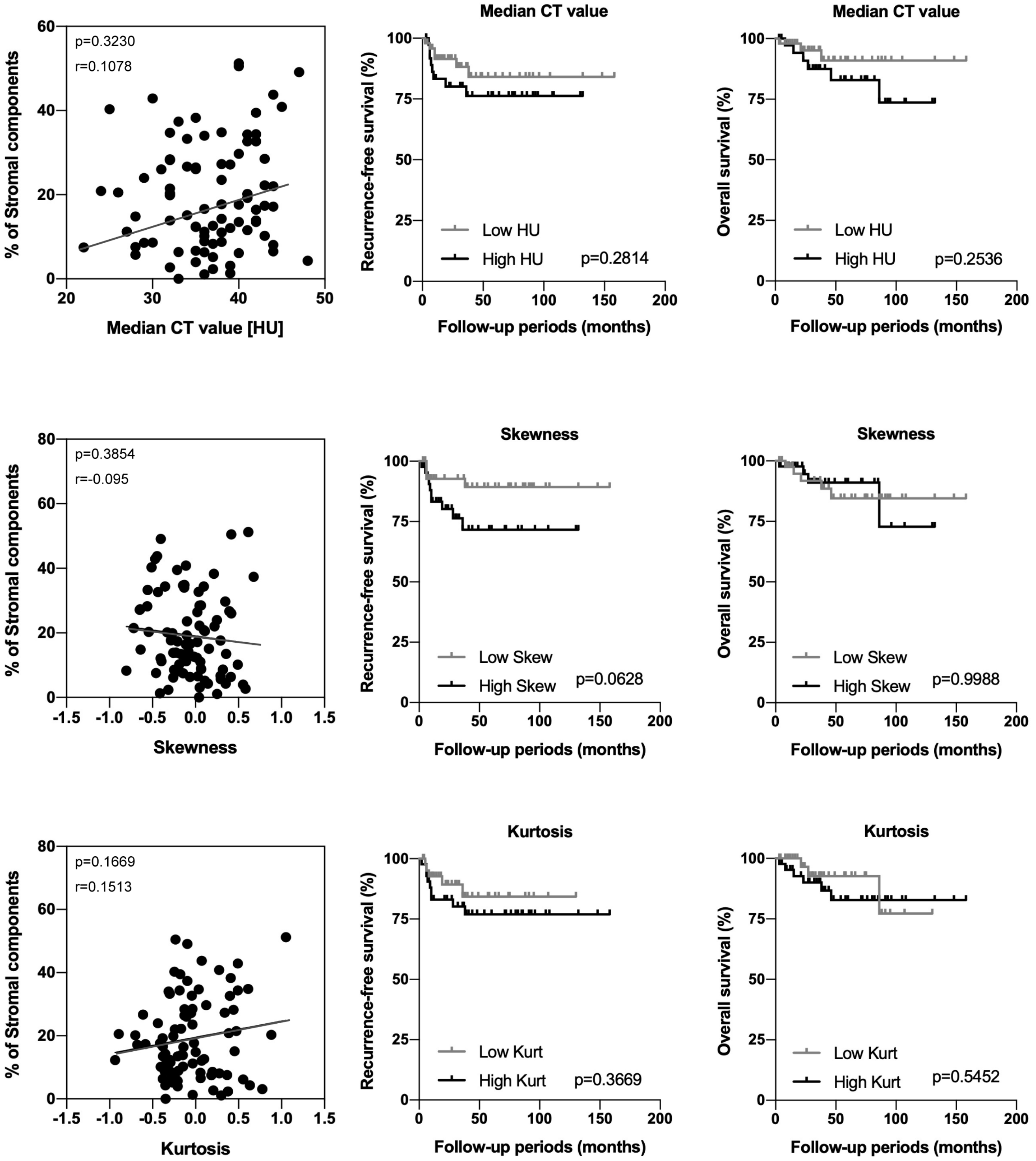

p=0.3230
r=0.1078
% of Stromal components
Median CT value [HU]
Median CT value
Recurrence-free survival (%)
Low HU
High HU
p=0.2814
Follow-up periods (months)
Median CT value
Overall survival (%)
Low HU
High HU
p=0.2536
Follow-up periods (months)
p=0.3854
r=-0.095
% of Stromal components
Skewness
Skewness
Recurrence-free survival (%)
Low Skew
High Skew
p=0.0628
Follow-up periods (months)
Skewness
Overall survival (%)
Low Skew
High Skew
p=0.9988
Follow-up periods (months)
p=0.1669
r=0.1513
% of Stromal components
Kurtosis
Kurtosis
Recurrence-free survival (%)
Low Kurt
High Kurt
p=0.3669
Follow-up periods (months)
Kurtosis
Overall survival (%)
Low Kurt
High Kurt
p=0.5452
Follow-up periods (months)

Figure S1

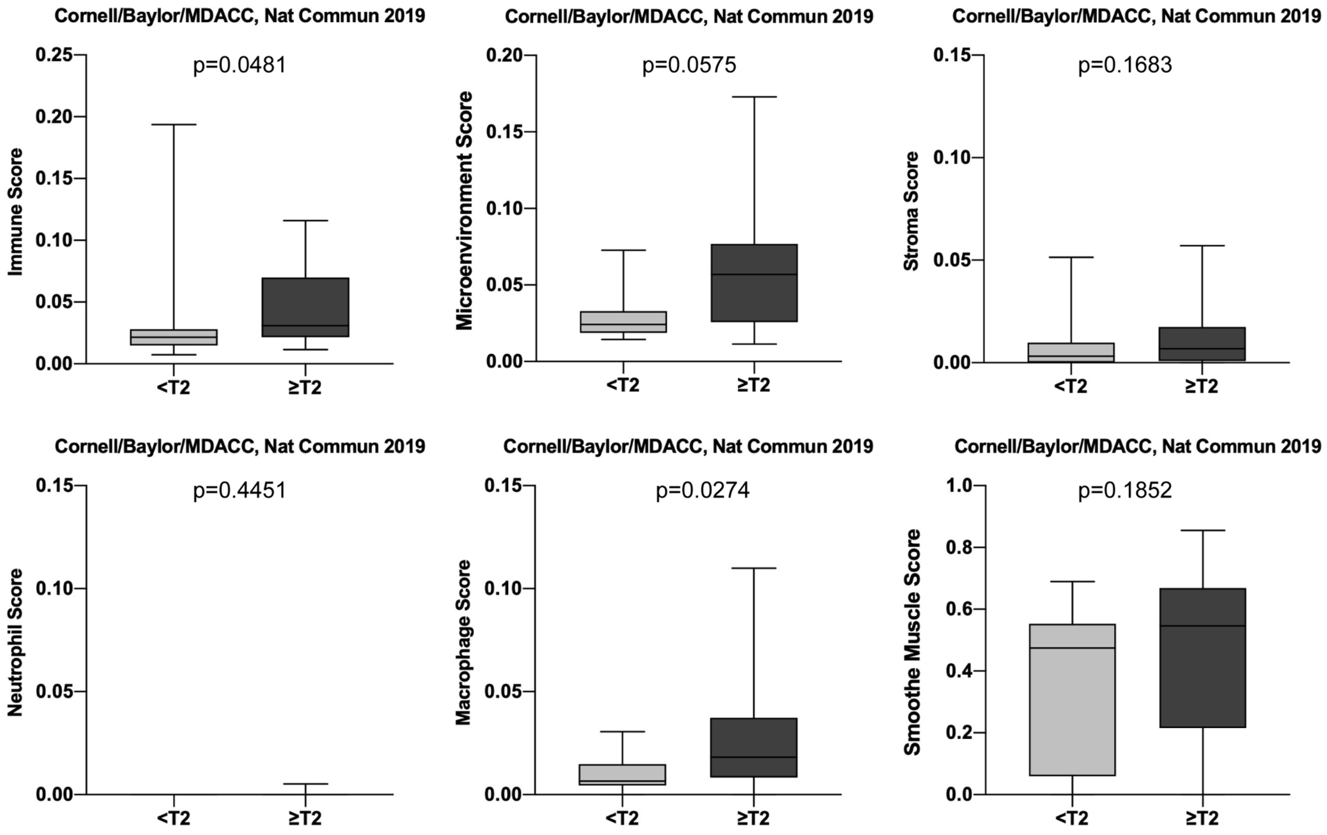
Cornell/Baylor/MDACC, Nat Commun 2019
p=0.0481
Immune Score
0.25
0.20
0.15
0.10
0.05
0.00
<T2
≥T2
Cornell/Baylor/MDACC, Nat Commun 2019
p=0.0575
Microenvironment Score
0.20
0.15
0.10
0.05
0.00
<T2
≥T2
Cornell/Baylor/MDACC, Nat Commun 2019
p=0.1683
Stroma Score
0.15
0.10
0.05
0.00
<T2
≥T2
Cornell/Baylor/MDACC, Nat Commun 2019
p=0.4451
Neutrophil Score
0.15
0.10
0.05
0.00
<T2
≥T2
Cornell/Baylor/MDACC, Nat Commun 2019
p=0.0274
Macrophage Score
0.15
0.10
0.05
0.00
<T2
≥T2
Cornell/Baylor/MDACC, Nat Commun 2019
p=0.1852
Smoothe Muscle Score
1.0
0.8
0.6
0.4
0.2
0.0
<T2
≥T2

Figure S2

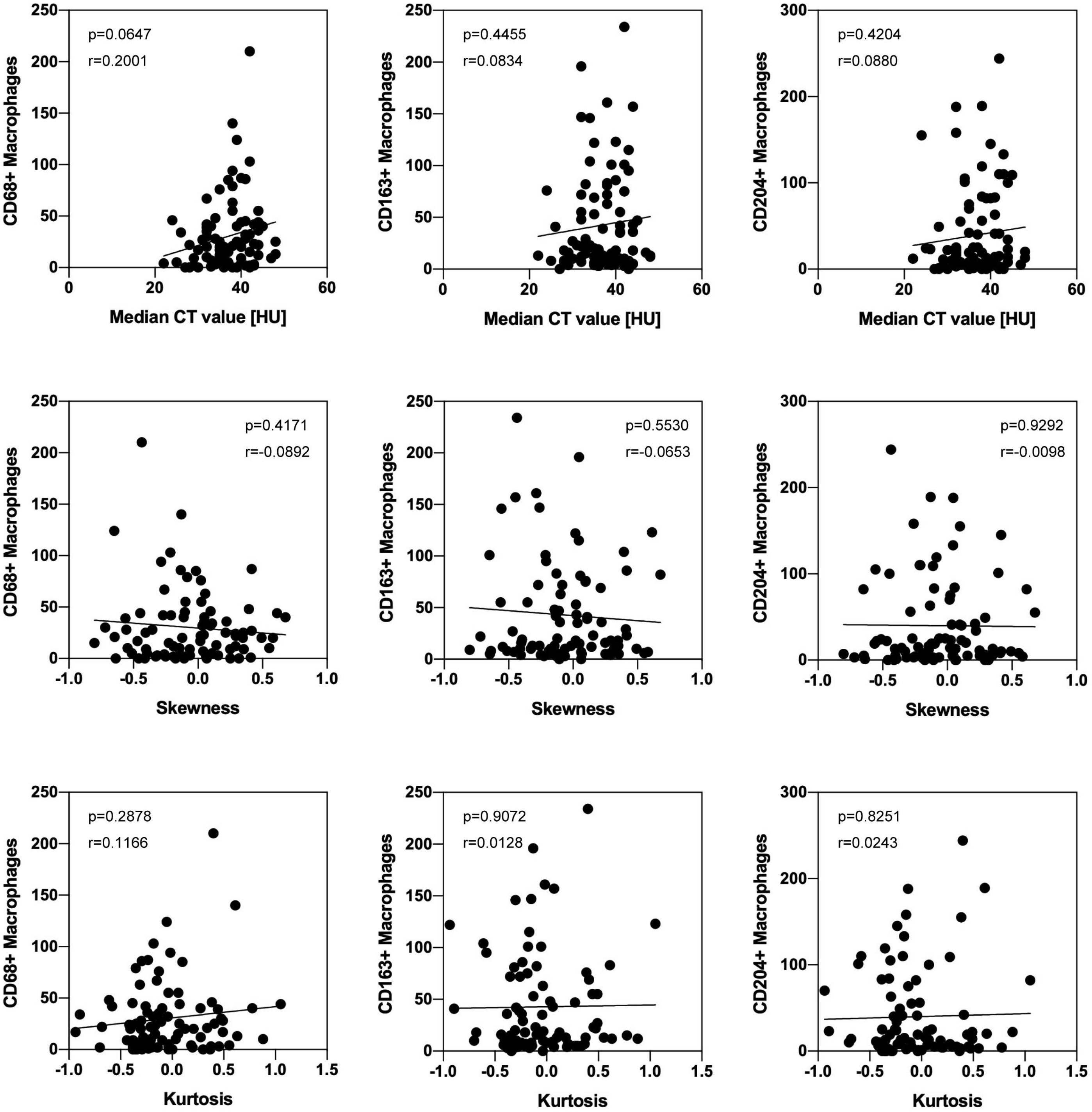
p=0.0647
r=0.2001
CD68+ Macrophages
Median CT value [HU]
p=0.4455
r=0.0834
CD163+ Macrophages
Median CT value [HU]
p=0.4204
r=0.0880
CD204+ Macrophages
Median CT value [HU]
p=0.4171
r=-0.0892
CD68+ Macrophages
Skewness
p=0.5530
r=-0.0653
CD163+ Macrophages
Skewness
p=0.9292
r=-0.0098
CD204+ Macrophages
Skewness
p=0.2878
r=0.1166
CD68+ Macrophages
Kurtosis
p=0.9072
r=0.0128
CD163+ Macrophages
Kurtosis
p=0.8251
r=0.0243
CD204+ Macrophages
Kurtosis

Figure S3

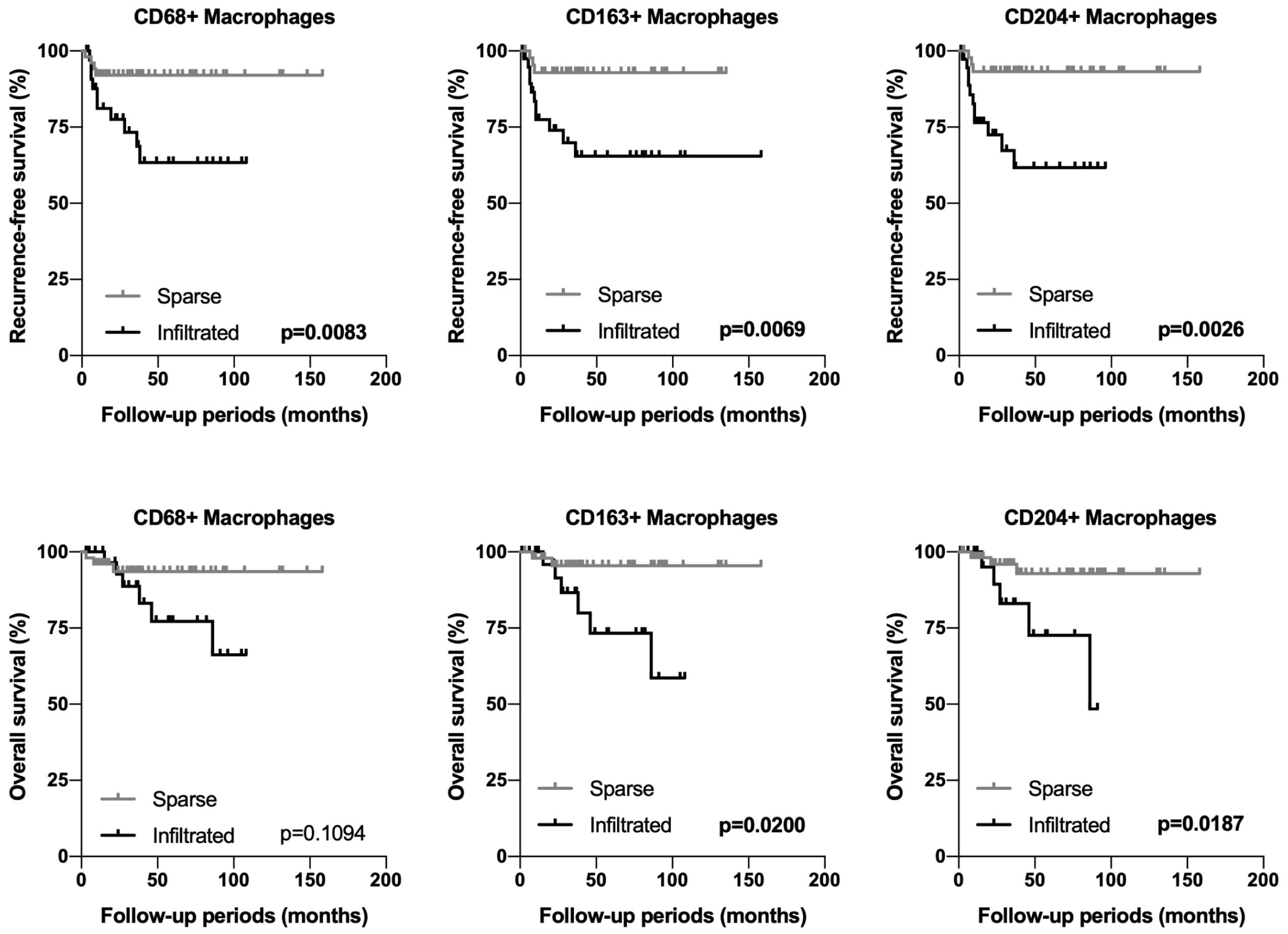
CD68+ Macrophages
Recurrence-free survival (%)
Sparse
Infiltrated
p=0.0083
Follow-up periods (months)
CD163+ Macrophages
Recurrence-free survival (%)
Sparse
Infiltrated
p=0.0069
Follow-up periods (months)
CD204+ Macrophages
Recurrence-free survival (%)
Sparse
Infiltrated
p=0.0026
Follow-up periods (months)
CD68+ Macrophages
Overall survival (%)
Sparse
Infiltrated
p=0.1094
Follow-up periods (months)
CD163+ Macrophages
Overall survival (%)
Sparse
Infiltrated
p=0.0200
Follow-up periods (months)
CD204+ Macrophages
Overall survival (%)
Sparse
Infiltrated
p=0.0187
Follow-up periods (months)

Figure S4

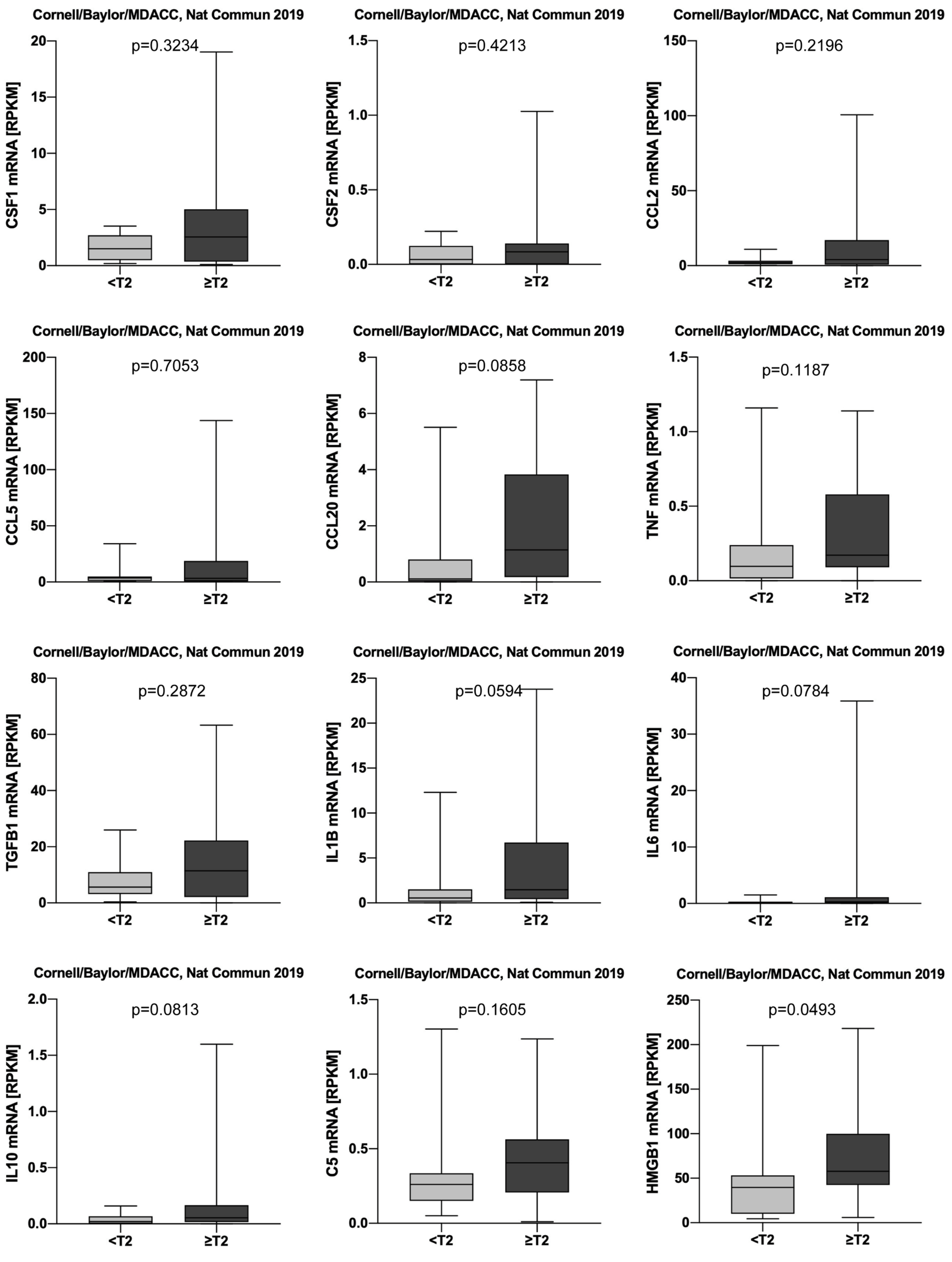

Cornell/Baylor/MDACC, Nat Commun 2019
p=0.3234
CSF1 mRNA [RPKM]
Cornell/Baylor/MDACC, Nat Commun 2019
p=0.4213
CSF2 mRNA [RPKM]
Cornell/Baylor/MDACC, Nat Commun 2019
p=0.2196
CCL2 mRNA [RPKM]
Cornell/Baylor/MDACC, Nat Commun 2019
p=0.7053
CCL5 mRNA [RPKM]
Cornell/Baylor/MDACC, Nat Commun 2019
p=0.0858
CCL20 mRNA [RPKM]
Cornell/Baylor/MDACC, Nat Commun 2019
p=0.1187
TNF mRNA [RPKM]
Cornell/Baylor/MDACC, Nat Commun 2019
p=0.2872
TGFB1 mRNA [RPKM]
Cornell/Baylor/MDACC, Nat Commun 2019
p=0.0594
IL1B mRNA [RPKM]
Cornell/Baylor/MDACC, Nat Commun 2019
p=0.0784
IL6 mRNA [RPKM]
Cornell/Baylor/MDACC, Nat Commun 2019
p=0.0813
IL10 mRNA [RPKM]
Cornell/Baylor/MDACC, Nat Commun 2019
p=0.1605
C5 mRNA [RPKM]
Cornell/Baylor/MDACC, Nat Commun 2019
p=0.0493
HMGB1 mRNA [RPKM]
<T2
≥T2

Figure S5

Supplementary figure legends

Figure S1
Scatter plots comparing CT attenuation number, skewness and kurtosis and the amount of stromal components and Kaplan-Meier analysis according to CT attenuation number, skewness and kurtosis.

Figure S2
Estimations of xCell score in 32 cases of UTUC (Cornell/Baylor/MDACC, Nat Commun 2019). Immune score, microenvironment score, stroma score, neutrophil score, macrophage score and smooth muscle score were compared.

Figure S3
Relationships between CT attenuation number, skewness and kurtosis and the number of CD68-, CD163- and CD204- positive macrophages. Correlations were evaluated using Pearson correlation coefficients.

Figure S4
Kaplan-Meier analysis according to the number of infiltrating CD68-, CD163- and CD204- positive macrophages. Recurrence-free survival and overall survival were examined.

Figure S5
mRNA expressions in 32 cases of UTUC (Cornell/Baylor/MDACC, Nat Commun 2019). Datasets were obtained from cBioportal. org (https://www.cbioportal.org). Genes involved in macrophage recruitment were analyzed.

Table S1.
Relationships between clinicopathological parameters and CD68-, CD163- and CD204-positive macrophages

	CD68+ macrophage	p-value	CD163+ macrophage	p-value	CD204+ macrophage	p-value
Hydronephrosis						
Mild	27.15 ± 31.21	0.3475	34.48 ± 38.24	0.0680	32.83 ± 46.22	0.1234
Severe	36.03 ± 43.71		59.78 ± 64.81		53.93 ± 62.22	
Urine cytology						
Negative	20.13 ± 21.67	**0.0127**	20.33 ± 32.63	**0.0009**	20.33 ± 32.63	**0.0009**
Positive	38.49 ± 42.72		56.09 ± 60.35		56.09 ± 60.35	
T stage						
Ta−T1	15.36 ± 18.08	**<0.0001**	18.27 ± 20.49	**<0.0001**	12.24 ± 16.66	**<0.0001**
T2−T4	49.24 ± 43.38		74.41 ± 57.56		75.49 ± 61.27	
Histological grade						
Low	14.21 ± 19.03	**0.0003**	13.45 ± 16.04	**<0.0001**	8.72 ± 10.59	**<0.0001**
High	37.95 ± 39.34		57.16 ± 53.78		55.09 ± 57.99	
Lymphovascular invasion						
Not evident	23.24 ± 30.88	**0.0208**	29.38 ± 34.68	**0.0030**	29.22 ± 43.71	**0.0193**
Evident	44.59 ± 41.08		70.93 ± 63.29		61.81 ± 62.72	
Concomitant CIS						
No	22.63 ± 24.96	**0.0200**	33.14 ± 42.36	**0.0135**	32.44 ± 45.78	**0.0414**
Yes	49.96 ± 50.65		67.83 ± 58.13		58.65 ± 64.40	

CIS= carcinoma in situ

Table S2.

Relationships between tumor heterogeneity evaluated by TA and CD68-, CD163- and CD204-positive macrophages according to each indicated pathological parameter

	Ta−T1 cases			T2−T4 cases		
	Standard deviation			Standard deviation		
	Low	High	p-value	Low	High	p-value
CD68+ macrophage	10.61 ± 11.41	23.56 ± 24.11	**0.0437**	43.75 ± 32.60	51.88 ± 48.10	0.5502
CD163+ macrophage	13.71 ± 12.07	26.11 ± 28.72	**0.0480**	71.83 ± 65.88	75.64 ± 54.55	0.8641
CD204+ macrophage	9.39 ± 11.53	17.17 ± 22.56	0.0932	71.25 ± 61.27	77.52 ± 62.43	0.7748

	Low grade			High grade		
	Standard deviation			Standard deviation		
	Low	High	p-value	Low	High	p-value
CD68+ macrophage	7.89 ± 8.01	24.55 ± 26.76	0.0347	28.48 ± 28.56	45.34 ± 45.12	**0.0457**
CD163+ macrophage	9.61 ± 6.43	19.73 ± 24.09	0.1002	44.56 ± 53.17	67.00 ± 52.98	0.0597
CD204+ macrophage	7.56 ± 11.47	10.64 ± 9.15	0.2163	40.40 ± 51.98	66.56 ± 60.61	**0.0427**

	LVI not evident			LVI evident		
	Standard deviation			Standard deviation		
	Low	High	p-value	Low	High	p-value
CD68+ macrophage	15.80 ± 20.54	34.08 ± 39.68	**0.0231**	37.63 ± 33.05	47.53 ± 44.52	0.5316
CD163+ macrophage	18.89 ± 20.59	44.67 ± 44.65	**0.0065**	78.25 ± 79.26	67.84 ± 57.53	0.7476
CD204+ macrophage	17.28 ± 28.27	46.63 ± 55.79	**0.0119**	67.63 ± 70.62	59.37 ± 61.00	0.7781

	CIS not evident			CIS evident		
	Standard deviation			Standard deviation		
	Low	High	p-value	Low	High	p-value
CD68+ macrophage	18.08 ± 23.08	28.32 ± 26.44	0.1122	27.63 ± 30.22	61.87 ± 56.01	0.0707
CD163+ macrophage	25.74 ± 41.02	42.39 ± 42.92	0.1242	48.25 ± 54.57	78.27 ± 59.04	0.2405
CD204+ macrophage	26.31 ± 44.91	40.11 ± 46.52	0.2400	28.13 ± 37.41	74.93 ± 70.72	0.0504

LVI = Lymphovascular invasion, CIS= carcinoma in situ

Table S3.
Relationships between HMGB1 expression and parameters obtained from texture analysis

	HMGB1 IHC		
	Weak	Strong	p-value
Median CT value [HU]	36.38 ± 5.72	37.32 ± 5.43	0.4580
Standard deviation (SD)	11.02 ± 3.57	13.80 ± 3.78	**0.0011**
Skewness	-0.073 ± 0.335	-0.040 ± 0.334	0.6636
Kurtosis	-0.054 ± 0.348	-0.042 ± 0.440	0.8933

HMGB1=high mobility group box 1, IHC=immunohistochemistry, CT=computed tomography, HU=Hounsfield Unit.
